# Understanding the economic impact of myalgic encephalomyelitis/chronic fatigue syndrome in Ireland: a qualitative study

**DOI:** 10.12688/hrbopenres.13181.1

**Published:** 2020-12-04

**Authors:** John Cullinan, Orla Ní Chomhraí, Tom Kindlon, Leeanne Black, Bláthín Casey

**Affiliations:** 1 School of Business and Economics, NUI Galway, Galway, H91 TK33, Ireland; 2 Irish ME/CFS Association, Dublin, Ireland; 3 Health Research Institute, University of Limerick, Limerick, V94 T9PX, Ireland

**Keywords:** Myalgic encephalomyelitis, Chronic fatigue syndrome, Economic costs, Thematic analysis

## Abstract

**Background:** Myalgic encephalomyelitis/chronic fatigue syndrome (ME/CFS) is a disabling and complex chronic disease of unknown origin, whose symptoms, severity, and progression are extremely variable. Despite being relatively common, the condition is poorly understood and routine diagnostic tests and biomarkers are unavailable. There is no evidence on the economic impact of ME/CFS in Ireland.

**Methods:** Adopting a patient and public involvement approach, we undertook three semi-structured focus groups, which together included 15 ME/CFS patients and 6 informal carers, to consider costs related to ME/CFS in Ireland, including how and why they arise. Focus groups were audio-recorded and transcribed verbatim, and we employed thematic analysis following the approach set out in Braun and Clarke (2006).

**Results:** Themes from the data were: (1) Healthcare barriers and costs; (2) Socioeconomic costs; (3) Costs of disability; and, (4) Carer-related costs. Patient participants described a range of barriers to effective healthcare that led to extra costs, including delays getting a diagnosis, poor awareness/understanding of the condition by healthcare professionals, and a lack of effective treatments. These were linked to poor prognosis of the illness by participants who, as a result, faced a range of indirect costs, including poorer labour market and education outcomes, and lower economic well-being. Direct extra costs of disability were also described, often due to difficulties accessing appropriate services and supports. Informal carer participants described a range of impacts, including time costs, burnout, and impacts on work and study.

**Conclusions:** The data suggests that ME/CFS patients face a wide range of costs, while there are also wider societal costs in the form of costs to the health service, lost productivity, and impacts on informal carers. These results will inform ongoing research that aims to quantify the economic burden of ME/CFS in Ireland and raise awareness of the illness amongst healthcare providers and policymakers.

## Introduction

Myalgic encephalomyelitis/chronic fatigue syndrome (ME/CFS) is a disabling, complex and often long-term illness ^1, 2^. Symptoms such as sleep disturbances, cognitive difficulties, pain, and orthostatic intolerance are common, though symptomatology, severity and disease progression are variable ^1– 3^. A defining feature of ME/CFS is an unusual response to even mild exertion, which can provoke a symptom complex known as ‘post-exertional malaise’ or ‘postexertional neuro-immune exhaustion’ ^2^. Routine diagnostic tests or biomarkers are not available, while the condition is generally not well understood by medical professionals ^4, 5^. As a result, many people with ME/CFS face considerable difficulties getting diagnoses, treatments, and supports ^2, 6^. Data from the United States (US) suggest about 90% of people with ME/CFS have not been diagnosed ^2^ and that a delayed diagnosis appears to be a risk factor for poor prognosis ^3^.

ME/CFS is a relatively common illness. Prevalence appears to be within the 0.2–0.8% range in developed countries ^7^, though this is highly dependent on case definition, while it is much more common in women. ME/CFS most commonly occurs between the ages of 20 to 40, but can affect all age groups ^6^. There are no study-based prevalence data available for Ireland, the context of our study, though patient organisations estimate there around 10–19,000 people with the condition ^8^. International estimates suggest there are around 250,000 people with ME/CFS in the United Kingdom (UK) ^9^, 2 million in the European Union (EU) ^10^, and up to 2.5 million people in the US ^2^.

As well as the direct ill-health consequences of the condition, which can be severely debilitating, there is extensive evidence that ME/CFS can also have a major impact on other aspects of people’s lives. A systematic review of qualitative studies found that illness development influenced identity, reductions in functioning, as well as coping ^11^. Indeed, research has shown substantial reductions in functioning for individuals across occupational, education, personal, and social domains ^12– 14^. For example, the condition can lead to the loss of social roles and major disruptions in personal relationships ^15– 18^.

Despite the large number of people affected by ME/CFS and the potential serious consequences for individuals, family members, and the economy, there is relatively little research examining the economic consequences of the condition. In fact, it is generally recognised that economic analyses of ME/CFS, including cost-of-illness (COI) studies and economic evaluations of interventions, are problematic due to the use of a variety of case definitions, as well as the unwillingness of many doctors to diagnose it ^10, 19^. This leads to a lack of accurate incidence and prevalence data, with no obvious way to estimate costs incurred by undiagnosed patients.

While there is some research on the economic impact of ME/CFS in countries such as the UK ^7, 20– 23^, the US ^2, 24– 26^, and Australia ^27, 28^, there has been no attempt to examine the issue in Ireland. In fact, there is very little previous research on the health and healthcare consequences for those affected in the Irish context ^5^. This is important, since one way of highlighting the need for greater awareness of a condition such as ME/CFS by medical professionals and policymakers, as well as improving services and supports for those affected, is through evidence on its economic impact, including the impact on healthcare utilisation ^19, 28, 29^.

In this context, this paper aims to better understand the range and nature of costs related to ME/CFS in Ireland. In particular, it seeks to elicit and analyse both patient and informal carer perspectives on these costs, including how and why they arise. In doing so, it adopts a public and patient involvement (PPI) approach and presents results from a study conducted by a patient-academic research partnership. Furthermore, in addition to being a novel qualitative research study that adds to existing literature by highlighting patient and carer perspectives, it also represents an important starting point in generating evidence on, and developing a comprehensive understanding of, the societal burden of ME/CFS in Ireland.

## Methods

### Study design

This is a qualitative study design using a semi-structured focus group methodology. A qualitative approach was adopted as it facilitates in-depth exploration and understanding of a variety of economic costs associated with ME/CFS for both patients and informal carers.

### Ethical approval

Ethical approval for this study was obtained from the NUI Galway Research Ethics Committee (Application Reference Number: 19-Aug-06). Written informed consent was obtained from all participants in the focus groups.

### PPI approach

This is research from an ME/CFS patient-academic partnership developed under the Community Engaged Scholars Programme (CES-P) at NUI Galway. CES-P is an education and training initiative that aims to increase the capacity of community-academic partnerships to work together to conduct research that is underpinned by principles of PPI, with the goal of improving the health and wellbeing of patients. In our partnership there are three patient (or PPI) partners (O.Ní.C., T.K., and L.B.) and one academic partner (J.C.). The overall goal of our partnership is to estimate the economic impact of ME/CFS in Ireland, and in this paper we use focus groups and qualitative research methods to develop a better understanding of the range and nature of costs that arise from the illness. Our results and findings will inform our future PPI-driven quantitative research.

As described in detail below, patient partners were actively involved in all aspects of this study, including: development of the questioning route (QR); design, organisation, and facilitation of the focus groups; data analysis; and, write-up of results. At all stages of the study, research-related decisions were made jointly by patient and academic partners.

### Developing the questioning route

A draft QR for this study was initially developed based on existing international literature in the area ^7, 10, 19– 28^, as well as the direct experience of the patient partners. In relation to the latter, this was achieved on the basis of a series of meetings between J.C. and O.Ní.C., who also consulted with other patient partners. Following discussions between the academic and patient partners, as well as an experienced qualitative researcher (B.C.), a final QR was prepared – see Table 1. It included sections relating to: Health and social care costs; Costs to individuals and the economy; Family members and carers; Welfare payments and supports; and, Other issues. A range of questions and probes for each section was included to allow for a semi-structured interview design.

**Table 1.** Questioning route.

Topic	Question(s)	Probe(s)
**Introduction** Researchers introduce themselves and the moderator. The researcher explains the purpose and format of the session. The researcher provides the group with ground rules for the duration of the focus group.	One at a time, can each of you say aloud your name and something about yourself? For example, my name is X and I am from Y. Let’s start to my right.	Where are you from?
**Health and social care costs** Researcher defines what is meant by health and social care costs.	When and how were you given a diagnosis of ME/CFS? What types of health and social care services do/have you use(d)? What types of treatments do/have you use(d)? What are the costs for your care and treatments? Have you faced barriers in terms of accessing treatments/ supports/services?	How long did it take you to get a diagnosis? Number of health care professionals you have dealt with? Are there good treatments available for you? Health care professionals understanding of ME/CFS? From where do you get information on ME/CFS?
**Costs to individuals and the economy** Researcher defines what is meant by costs to individuals and to the economy.	Has ME/CFS impacted your ability to work? Has ME/CFS impacted your education? Has ME/CFS impacted your standard of living?	Employment situation prior to ME versus now? Reduced hours? PT/FT? Left job? Missed out on promotion? Early retirement? Impact on future prospects? Employers/educators knowledge of ME/CFS?
**Family members and carers** Researcher describes potential costs that can arise to family members and informal carers.	How much time do you give to caring activities? Has caring impacted on your ability to work? Has caring impacted your own health and wellbeing?	What aspects of caring for someone with ME are challenging?
**Welfare payments and supports** Researcher discusses welfare payments and supports.	What supports (financial or otherwise) do you receive? Any issues accessing supports?	Have you applied for social welfare payments as a direct result of your ME/CFS?
**Other issues** Have we missed anything?	Any other relevant costs?	Do you face extra costs of living due to ME/CFS?
**Conclusion** Plan is to use the findings from these FGs in future research. We are thinking about undertaking a national study on the burden of ME/CFS and interested in your views on how best to do this?	What are your thoughts on such a study? Would you be willing to complete a potentially long survey questionnaire and how?	Mail v online v face-to-face? Interviews better?

### Participants

ME/CFS patients and informal carers were invited to take part in this study by members of the research team (L.B. and O.Ní.C.). Participant selection was conducted through several different avenues in an attempt to get a diverse sample population in terms of age, length of diagnosis, sex, and geographical location. Potential participants were invited through the Irish ME/CFS Association and the Irish ME Trust, as well as through the ME Ireland Facebook group. For example, an invitation to participate was included in the November 2019 edition of the Irish ME/CFS Association Newsletter. Snowballing techniques such as ‘word of mouth’ and sharing of social media recruitment content were also used. Once potential participants were identified, they were given a study information sheet and consent form. Potential participants had two weeks to read the study information sheet and return the consent form to the research team via email or phone. Individuals were considered eligible if they were (i) a patient with ME/CFS; or, (ii) an informal carer of a patient with ME/CFS. Inclusion criteria were:

1. Patient diagnosed with ME/CFS by a medical professional;
2. Informal carer providing unpaid care to a family member or friend diagnosed with ME/CFS;
3. Primary condition is ME/CFS;
4. Aged 18+ years.

Exclusion criteria were:

1. Primary condition is not ME/CFS;
2. Patient has not been diagnosed with ME/CFS by a medical professional;
3. Professional or paid carers providing care to a person diagnosed with ME/CFS;
4. Aged <18 years.

Representatives from the Irish ME/CFS Association (O.Ní.C., T.K., and L.B.) acted as gatekeepers for recruitment of known participants. Interested applicants were advised to contact the academic partner (J.C.) or a patient partner (L.B. or O.Ní.C.) for further information. Focus groups were organised once the research team had recruited patients with a range of experiences of the illness and data saturation was reached.

### Setting

There were three focus groups held in three cities across Ireland (Galway, Dublin, and Cork), which were chosen for convenience sampling purposes and to provide a good geographic spread. The focus groups were conducted in November and December of 2019 and each focus group was organised to take place in late afternoon or at evening time to facilitate ME/CFS patients, who often have difficulty attending at earlier times as a result of sleep problems. Each focus group lasted for 2 hours and included breaks and refreshments as required. The first focus group was co-facilitated by J.C. and O.Ní.C. and the second and third were facilitated by J.C. only. Each focus group was audio-recorded, while an assistant moderator (B.C. or L.B.) recorded group dynamics to supplement findings within the thematic analysis described below. Participants were invited to submit additional comments to any member of the research team at a later date if they wished to do so.

### Data analysis

We followed the approach of Braun and Clarke (2006) ^30^ to undertake thematic analysis of our focus group data. This involved six phases, namely: familiarisation with the data; generation of initial codes; searching for themes; reviewing themes; defining and naming themes; and, producing a report. Thematic analysis was chosen as it is flexible in its approach, while also providing a rich, detailed, but yet complex account of the data ^30^. In addition, investigator-led triangulation was employed throughout the described thematic analysis ^31, 32^. The involvement of an economist (J.C.), a person living with ME/CFS (O.Ní.C.), as well as an experienced qualitative researcher (B.C.), aimed to bring confirmation of findings and different perspectives on the data presented.

In step 1, data were transcribed verbatim by a professional audio transcribing services company. Errors were checked within each transcript against the tape recording and all transcripts were read several times (J.C. and O.Ní.C.), with initial ideas noted. Step 2 involved the generation of initial preliminary codes, which were drawn from keywords and phrases related to the research question and from discussion between two authors upon reading and re-reading the transcripts (J.C. and O.Ní.C.). To promote rigour, two authors (J.C. and O.Ní.C.) read and re-read each of the three transcripts, from which they devised the coding list which was compared, discussed and finalised. Notes were taken to document key decisions.

In step 3, authors searched for themes within the data. All text was coded using the coding list developed in step 2 (O.Ní.C.), and codes were then checked for consistency (O.Ní.C. and J.C.). Similar codes were grouped together into subthemes, which were then examined to identify how these might form main themes from discussion between two authors (O.Ní.C and J.C). In step 4, themes that were identified from clustering of subthemes from grouped codes in step 3 were reviewed (O.Ní.C., J.C. and B.C.) and the authors checked that themes worked in relation to both the coded extracts, the entire data set, and the research question.

Step 5 involved defining and naming themes in which development of a diagram to represent the themes also occurred. Step 6, the final step, involved selecting quotes from the raw data to best capture themes and subthemes to write-up the results for this paper (O.Ní.C. and J.C.). Reflexivity was promoted by taking field notes during data collection and throughout the analytical process to track opinions and beliefs regarding the data ^33^. This was undertaken by both academic (J.C) and patient researchers (O.Ní.C. and L.B.), under the guidance of an experienced qualitative researcher (B.C.).

## Results

There were a total of 21 participants across the three focus groups. This included 15 ME/CFS patients and 6 informal carers. The sample was almost entirely female (n=20), though 4 of the carers were caring for male patients. The sole male participant was an informal carer.

**Figure 1.**
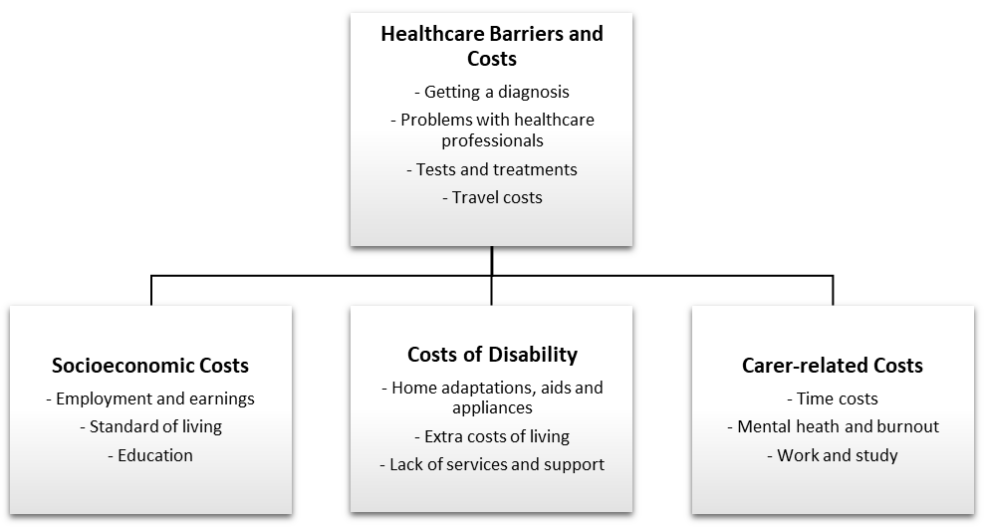
Economic impact of myalgic encephalomyelitis/chronic fatigue syndrome.

The findings of the analysed data are presented in Figure 1. Four themes and associated sub-themes are depicted. These themes and a selection of quotes to illustrate the sub-themes are presented in the next four sub-sections. Importantly, the barriers to appropriate and effective healthcare identified in the first theme also have important implications for each of the other three themes, in terms of increased socioeconomic costs, costs of disability, as well as carer-related costs, and this relationship between the themes is reflected in Figure 1.

### Healthcare barriers and costs

This theme represents the barriers and costs faced by patients in accessing and utilising effective healthcare services. Subthemes of the data include: getting a diagnosis; problems with healthcare professionals; tests and treatments; and, travel costs.

***Getting a diagnosis.*** Participants described a range of problems and costs that related to getting a diagnosis of ME/CFS. For some it took years, with numerous visits to GPs, consultants, and other healthcare professionals for their illness to be identified or even acknowledged. Participants highlighted how they were often passed from one healthcare professional to another. In many cases, consultations to get a diagnosis were paid for out-of-pocket, at significant personal cost.

“I was sent from one consultant to another consultant. … One would check me out, no, you’re all right there, send me to someone else. I think I went to four or five. I ended up with the immunologist, who diagnosed me then in 2010. … Three years from one person to the other.” **P11**

A recurring issue related to getting a diagnosis was a refusal of some healthcare professionals to provide a diagnosis. In some cases, this stemmed from disbelief about the condition, in others from a general lack of understanding and awareness. In many cases, this led to significant costs for patients.

“And despite me saying to him (GP), well you know the World Health Organisation have recognised it as a disease, are you telling me that you don’t believe it exists, and he (GP) said yes, I don’t believe. He said with your son, I’d like to start again. So, anyway, he’s on a waiting list for [an Irish] diagnosis.” **C1**

There were also specific issues relating to the diagnosis of children. Again these related to both a general lack of willingness to diagnose the condition, as well as a misunderstanding and lack of awareness of the nature of the condition. One carer participant mentioned that neither of her two children, who both got sick when they were 12 years of age, could get a diagnosis until they turned 18 years of age. Participants also described how children, in particular, were sometimes simply not believed.

“I got a virus when I was 16 and it took two years before a locum GP diagnosed me as Chronic Fatigue [Syndrome]/ME but at that time my GP just kept telling my parents that I was lazy. Looking for attention, she's the oldest of four she's looking for attention and that’s all it is, ignore her.” **P13**

***Problems with healthcare professionals.*** Disbelief and a general lack of understanding and knowledge about ME/CFS were also factors that led to costs after being diagnosed. Participants described a wide range of serious problems they experienced with healthcare professionals and services in this regard. For some patients, finding an understanding healthcare professional was a significant challenge, often spending considerable time and resources finding appropriate care and/or on waiting lists for private care. For those who were more successful in finding suitable healthcare, it often meant paying extra.

“I stick with the same doctor now because she's good for me and she believes in what's happening with me. So … because she's a consultant, I'm paying [more] for my GP visits than I would be paying with a regular GP, but it's not an option for me to move to a GP and pay the lower rate because they're just, they're just not out there that they understand what you're going through.” **P15**

Other patient participants described how their doctors either had little interest in ME/CFS, didn’t really believe in it, or were completely unaware of the condition.

“I was put on meds for my skin and I had an allergic reaction and I ended up in A&E in [Hospital] X. And I had three doctors that not one of them even knew what ME was. … Like one doctor, she actually went and looked it up. She says, ‘I’ve never heard of that before’. And that is in Hospital X.” **P11**

The mistaken belief that ME/CFS is a psychological or psychiatric condition by many healthcare professionals was identified as a particularly important issue by participants. In some cases, this led to inappropriate treatment and care and the utilisation of scarce mental health services and resources. Some participants described how they faced direct financial costs themselves as a result of this misunderstanding.

“But when I got the diagnosis with the immunologist, they only said, ‘Right, we’re done with you. We have discovered this is what you have’. They said CFS. ‘We’re sending you to the psychiatrist’. And it’s gas. Even my psychiatrist that I went to, he even said to me like, … ‘They’re telling me it’s your body that’s letting you down, but they send you to a head shrink’. Like it’s contradictory. … Like he said it to me. He said, ‘It’s … contradictory’. And I said, ‘Well, I know’.” **P11**

In other cases, a view was expressed that patients should be ignored.

“I have had a lifetime of there's nothing we can do, oh that's not my area, until I got sick in 2008 when I had the full blown ME and all of a sudden it became, oh, we'll send you to psych and psych are just like going oh yeah, you need a course of CBT and you need to get exercising and you need to want to live life. The newest thing I've got from psych here is, he told my family I just didn't want to be involved in life and when I, that until I decided I wanted to live, just leave me in the corner and for everyone else to get on with their life” **P13**

Carers of children with ME/CFS noted that young people were particularly vulnerable to a psychiatric misdiagnosis, often leading to significant financial and other costs.

“But the cost was very much emotional as well as monetary because they tried to convince me that my children were depressed, that they had mental health issues. I had to pay to have them seen by a psychologist privately to prove that they weren’t and I had to refuse CAMHS from even coming to the house because I was afraid that they were going to take my second kid away.” **C6**

Participants also described how problems with healthcare professionals and services, including the mistaken view that ME/CFS is a psychological/psychiatric/behavioural disorder, or being provided with inappropriate care, exacerbated their condition, leading to increased costs.

“When X got the glandular fever, he was immediately sent to a consultant in Hospital X, … and looking back, that’s six years ago, and he was put on antidepressants straightaway and made do cognitive behavioural therapy. And like that frustrates me so much because I wonder if those … eight months of it, where he had to walk a certain distance every day, he had to do this, that and the other, and now knowing the research that he’s done on this, like it’s the worst thing that you could do. Now X weaned himself off antidepressants, I remember him coming in one day and saying, Mum, I can’t take this anymore, I can’t feel anything. And he did, he just weaned off, he wasn’t depressed.” **C5**

As a result of lack of awareness, disbelief, misdiagnosis, and financial costs, participants described how they stopped informing healthcare professionals about their condition, while others limited visits to their doctor or ceased seeking medical care.

“… then there's the trips to the GP, which I don't do very often, because she's so dismissive.” **P5**

***Tests and treatments.*** Participants described considerable private costs relating to both tests and treatments. The considerable financial burden arises both due to the number and range of tests undertaken and variety of treatments that are tried, as well as the considerable expense of individual tests and treatments. Often these are private costs paid for out-of-pocket.

“Yeah, it’s cost an absolute fortune in terms of treatment, in treatment costs. I’ve tried absolutely everything.” **P3**

Given the lack of understanding of many healthcare professionals, as well as the lack of effective treatments or a cure for ME/CFS, many participants turned to alternative therapies, often at considerable personal expense, since these are not generally covered by public or private health insurance.

“When you go for a diagnosis, first you meet the doctor who either doesn't know anything about it or who doesn't want to know. So you go and to try other treatments.” **P7**

Participants described consultations with acupuncturists, dieticians, reflexologists, natural healers, holistic practitioners, and others, following poor outcomes and experiences with more traditional health care professionals and treatments. The financial impact of this was described as “ridiculous”.

“Like X, I would have tried an awful lot of the alternative therapies as well, … and I went through a whole rake of different physiotherapists … the alternative stuff and it's costly because it's not a one off is it? You're going every single week for months or years and … all you're doing is managing, … you're never extinguishing the fire, you're just keeping it a little bit tame.” **P15**

Despite the considerable expense, there was an acknowledgement and realisation amongst participants that most tests and treatments were of limited effectiveness, despite being very costly. So while participants were spending considerable amounts of their own money on a variety of treatments, there was only limited benefit from this expenditure.

“… and there’s nothing conclusive to any of these, there’s nothing really conclusive. There’s no definitive thing that’s going to make this go away really, so it just all costs a lot of money.” **P3**

***Travel costs.*** Participants described a range of travel-related expenses associated with diagnosis, tests, and treatments. This included direct travel costs such as car journeys and rail fares, as well as overnight stays due to difficulties caused by ME/CFS in making trips in a single day. Often a carer was also required to travel at additional cost.

“I see another doctor now in X so I'm up and down every month to a consultant up there … So I go up on Friday, we'll stay in a hotel, my husband has to take the day or a night off because he works shift work, we'll stay in a hotel the night before and then I'll go to the doctor and come back because the round trip is too much.” **P9**

For some, these travel costs were incurred within Ireland. For others, travel costs arose due to trips abroad for medical care.

“So, in the meantime about a year and a half ago, we went and got Dr. X, who’s a UK consultant. So, we’d to go to X. We flew over, saw him and immediately [he] diagnosed that’s what it was.” **C5**

### Socioeconomic costs

This theme relates to the impact of ME/CFS on a range of socioeconomic outcomes, and includes impacts on individuals, families/households, and wider society. Subthemes include: employment and earnings; standard of living; and, education.

***Employment and earnings.*** For many patient participants, ME/CFS had a major impact on their careers, often with devastating implications for their current and future employment prospects, as well as for their earnings/income. A number of patients spoke about the considerable challenges they face in relation to working due to the specific nature of the condition, e.g. brain fog and post-exertional malaise. Others described how even if they could work, it was often only for very limited amounts of time.

“My GP knew me and … knew me to be an extremely healthy, energetic, vibrant, well person. So that [ME] was diagnosed and I stopped work then. I was … almost bed-bound for two years and almost house-bound for about 10 years after that. … At the moment, I’m just on a bit of a dip, so it’s probably two mornings a week for an hour each morning [that I work].” **P4**

Participants described how ME/CFS had forced them to retire early and the financial impact of that. In some cases, early retirement was a direct consequence of the severity of the illness, while in other cases issues with employers meant that respondents faced little choice other than to give up their jobs.

“I went out on sick leave and I was out for three years [due to ME], which was the maximum that you could stay out and so my place of work insisted that I leave but, like X, they didn't want to sign off on it, so they wanted me to do the retiring so I was forced into doing it and so I left and that was five years ago now. So I left with half of the pension that I would have been entitled to for all of my years of service. … [A] huge financial cost.” **P7**

Reduced employment possibilities and early retirement had serious financial implications in the form of reduced earnings and income for participants. Patients spoke of considerable income losses and the challenges this brought.

“Loss of income is phenomenal. … I've lost an annual income of around about 100k a year, I haven't been able to return to [my job] for several years now. I tried to reduce my hours, I tried to work part-time, eventually had to give up. So financially the implication is huge.” **P3**

“I had like a six-figure salary. I was, you know, at the top of my career and now I’ve nothing. … You know, all that money that I had is gone … We sold the car, re-mortgaged the house, you know.” **P1**

As well as the devastating impact on individuals’ own personal socioeconomic circumstances, participants also discussed the economic losses to wider society arising from ME/CFS. A number described the contribution they felt they made to society through their work and careers prior to onset and how this has been lost. In particular, it was noted by a number of participants that the benefits of their education and training would not now be realised, either for themselves or more widely.

“So on a personal level I've lost that level of income, on an economic level is the amount of tax that I'm not paying on that kind of income. … Yeah it's massive in terms of the eight years it took for my training to become professionally qualified, … in terms of the investment from the state into my training.” **P3**

***Standard of living.*** Not surprisingly, given the impact of ME/CFS on labour market outcomes, participants also described how ME/CFS has had a major negative impact on their economic well-being and standard of living. As well as leading to reduced employment and income, many patients were forced to use their savings and pensions contributions.

“I'm thinking [of] the long term expense, like previously if I hadn't gotten sick I had a very good pension, I had money that I put aside for retirement. That's all gone now, my pension is pretty much zero.” **P9**

The implications of these diminished financial circumstances have resulted in a much lower standard of living for respondents. For some it has meant forgoing basic items, for others a precarious financial situation and/or problems with debt.

“I'm sure I'm probably not alone in this but from a financial perspective I have over the years had to make choices between whether I get oil for my house or whether I get a treatment and there have been times I've picked the treatment and I'm sure I'm not alone in that… So I'm, I suppose I'm stuck in a cycle of debt [because of ME], so I feel like kind of trapped in so many ways financially.” **P15**

Respondents also spoke of how all of this had led to a much lower quality of life. For many it was a direct result of the chronic nature of ME/CFS, exacerbated by the financial and economic consequences of the condition.

“There’s days where there’s no one in my house that I wouldn’t eat all day because I can’t get out of the bed to even go down and get a glass of water. And it really, you know, cuts right to your core, your self-esteem, your self-worth, you know. And it’s a struggle every single day.” **P11**

***Education.*** A particularly important issue for younger participants and carers of children and younger adults was the impact of ME/CFS on educational experiences and outcomes. Participants and carers described how ME/CFS made participation in education extremely challenging. This was true both at secondary and third level, as well as in academic and social terms.

“With my two kids they both got sick between say first year and second year of secondary school and things went downhill very quickly. For example, with X, two hours of school would lead to three weeks in bed completely but the pressure would still be on for him to go in every three weeks for that two hours so that he could go back to bed for three weeks and it was a horrible cycle.” **C6**

The inability to participate fully in education had negative implications for educational attainment and outcomes. Participants described how completing the Leaving Certificate exams was either not possible or extremely difficult, while others noted how ME/CFS impacted on performance in college.

“Yeah X would have had two years done in University when he got sick, … so he had to take two years off. Then he went back and … College X has been pretty good in allowing him to study each year, over two years and so he's been six years in college now, but September just past there he would have been due to go into his final year, fourth year over two years and he can't. He's back at home, he hasn't left the house in four months now.” **C5**

### Costs of disability

This theme relates to costs incurred by patients with ME/CFS that are related to the disabling nature of their condition and their need for services and supports. These are additional costs that would not ordinarily arise for most people and can be one-off costs or costs arising in day-to-day living. Subthemes include: home adaptations, aids, and appliances; extra costs of living; and, lack of services and supports. Again these costs are generally higher the more severe and debilitating the illness, which can be related to some the issues described in the first theme.

***Home adaptations, aids, and appliances.*** Participants described how it was necessary to make adaptations to their homes as a direct result of their illness, often at considerable financial expense. A particular need arose in relation to downstairs bathrooms. Participants living in two-storey dwellings described how it was not possible for them to easily transition between upstairs and downstairs and how the lack of a downstairs bathroom meant they were constrained in terms of their living arrangements. As a result, significant renovations were required for a number of participants.

“Last year we had to put in a bathroom downstairs to use and we also live in a two storey house but I wasn't able to get up over the stairs for the bathroom and one day I tried and fell over, … so that was that, there was no-one there, so we needed a downstairs bathroom. So at least now once I make it downstairs I can stay there for the day.” **P9**

Participants also described how they faced additional expenditure as a result of necessary aids and appliances. In some cases it was possible to access these through the Health Service Executive (HSE) at no personal expense, but in other cases participants did face significant out-of-pocket costs on such items. Items like mobility devices were identified by a number of participants, while others noted items they required, such as a small freezer box by their bed, which were needed due to living alone and being unable get out of bed at times.

“Somebody else was mentioning aids that you'd use like the scooter or the wheelchair or chair, those kind of things, that's more a long term [item] you'd get one every so often if you could afford it and yeah, there'd be lots of costs with that.” **P12**

***Extra costs of living.*** As well as once-off or irregular expenditures, participants also described a range of extra costs of living they faced as a result of ME/CFS. For example, participants described extra costs incurred on items used by everyone but which people with disabilities tend to use more often. Additional heating costs, cleaning, and childcare services, as well as the need for online shopping services, were noted by participants.

**P9**     “One thing with me is my temperature control of my body. I get cold so easily if my house is not 22 degrees …

**P15**     … yep …

**P9**     … I'm freezing, and I mean, I'm X [nationality], I'm used to the cold, but it has to be at 22, as soon as it hits 21, I'm absolutely in pain …

**P15**     … yeah …

**P9**     … blue I can't do anything, can't move so it's that, that's a huge cost of the amount of coal and oil that we go through on a yearly basis. It's not normal by no means but there is no option it has to be done and …

**P5**     … yes I have that as well …

**P9**     … yeah you're the same yeah.”

There were also costs noted by participants that were related to specific symptoms or effects of ME/CFS, e.g. paying for home visits from medical professionals, such as dentists or chiropodists, because of being house-bound or bed-bound. One participant also described how their brain fog and memory issues often led to additional costs.

“Yeah I find my memory is a huge problem. … and my concentration, it creates a lot of unnecessary kind of mistakes [like forgetting to pay a fine], mistakes I'd never make if I wasn't sick. … You just forget, it completely goes out of your head and before you know it, the fine is double.” **P15**

There were also specific additional costs of living that arose for participants who were living alone and those who were employing a personal assistant (PA).

“If you're employing your own PA's which, although … the money coming from the HSE covers the carer hours, it doesn't cover the paperwork, it doesn't cover the, you know having to have a laptop and printer and the printer ink so that I can do the timesheets, it doesn't cover the scooter, it doesn't cover the scooter insurance, it doesn't cover the car and the car insurance and, and, and, and then all the health and safety things, trip hazards and so on, fire stuff, because you have employees in your own home. So all that is additional cost.” **P10**

***Lack of services and supports.*** A particularly problematic issue for participants was a lack of appropriate services and supports. Difficulties accessing basic care services were described, along with the costs this brought and the negative impact it had on quality of life.

“A lot of people can't actually access any help from the HSE so if you need any help you have to … hire them yourself. … Yeah I've been turned down for a PA and for a home help because the home help’s saying I should be getting a PA and PAs say I should be getting home help.” **P2**

Participants also described considerable difficulties they had in terms of accessing social welfare supports and payments. Often this was directly related to a basic lack of knowledge about ME/CFS.

“And I remember handing her the leaflet and I saying this is what ME is and this will explain to you, you know, the symptoms and what people with ME go through and why it’s difficult for people to work. And she looked at the leaflet and put it aside and she says, ‘I don’t need that’. … I said, ‘If you don’t understand the illness that I’m going through, how can you make an informed decision on whether I’m fit for work or not?’” **P11**

Difficulties accessing basic and necessary services and supports were not limited to care services and social welfare payments. Participants also described a wide array of issues and problems they faced in relation to accessing help for education, employment, and housing. The latter, in particular, was identified as particularly problematic for some participants.

“My children's access to education was incredibly limited.” **C6**

“When I contacted the local Council and told them of my inability to find suitable housing, … they referred me to the local homeless shelter. … That that’s where I should live.” **C6**

Another notable issue relating to supports and services was the high levels of bureaucracy or administrative burdens faced by participants. Participants described the considerable efforts they had to go to in order to explain what ME/CFS was/is to receive supports and services. Others described additional barriers they faced, particularly in relation to accessing education supports.

“You shouldn’t have to prove that CFS or ME exists. … We shouldn’t have to go through all of these hoops.” **P1**

### Career-related costs

The fourth theme relates to costs incurred by informal carers and family members of patients with ME/CFS. These costs are not just financial in nature but also include impacts of ME/CFS on carers’ day-to-day lives, their psychological well-being, as well as socioeconomic outcomes. Subthemes here include: time costs; mental health and burnout; and, work and study.

***Time costs.*** Both carer and patient participants described the often very long hours spent caring for a person with ME/CFS, particularly those most severely impacted by the illness. Some carers described how they had been providing informal care for extended periods of time, while some patients noted their informal carers did not receive any financial compensation from the State for the role they fulfilled.

“So it's [caring] been 24/7 for 12 years until X [a friend] persuaded me to take a break in September. So I took my first few nights away in 12 years.” **C6**

Participants described how informal caring had a major impact on the day-to-day lives of many carers. Caring placed constraints on what carers could do and how they spent their time, while some carers moved homes and/or locations to fulfil the role.

“I'd prepare her breakfast and do lunch, leave it there and sometimes I would come home from work and give her her lunch. I was also a member of the X and it [caring] meant, and it does mean, that if I go anywhere I have to be back in the evening to make sure that, as I said, I can't stay out overnight anywhere [because of caring].” **C3**

***Mental health and burnout.*** Participants described the often considerable impact of informal care responsibilities and demands on carers’ mental health, which in some instances led to considerable psychological stress and burnout. The efforts involved in providing informal care also had negative impacts on relationships.

“Myself, over the years, … I have been depressed at times over it, and it has affected our relationship and it's been very difficult.” **C2**

“I was, I'd say, a fraction from total burnout.” **C1**

Participants described how part of the mental health difficulties they faced are a result of the general disbelief around ME/CFS and the need to constantly fight for recognition of the condition.

“I would think the cost of mental health would be huge in trying to constantly fight for them and fight for their rights and never giving up on them because I deep down knew that there was something wrong that wasn’t mental, that it was a physical difficulty. … It’s a huge cost on my health too.” **C6**

Carer participants described feeling both helpless and hopeless, given the lack of available treatments and cures for ME/CFS. Some mentioned feeling like they were on their own when dealing with the condition.

“It has been very, very difficult and the big thing I found was both being helpless and feeling kind of hopeless that I should be able to fix something and I can't.” **C1**

***Work and study.*** Patient participants discussed how their informal carers ended up taking time off work to care for them, while carer participants described how they had to take early retirement or were unable to go back to work. In general, there were significant financial implications associated with these adverse labour market outcomes.

“I took earlier retirement … I thought he was deteriorating, … I didn't know how to treat him but then I felt I couldn't keep going those 12 hour shifts and come home. I thought that if I didn't stop working that I didn't know where this was going. … So … it has affected my pension because I didn't have enough years in, it affected certain incomes [that] have come in since then, [it] has affected all that.” **C1**

In other socioeconomic outcomes, participants described how their caregiving spouse had to take on an additional job due to the financial pressures they faced as a result of ME/CFS, or how carers’ educational outcomes and careers had been negatively impacted.

“My husband had to take on a second job and even [at] that we’re still struggling to pay our mortgage and to keep ourselves above water, pay our bills and, you know.” **P11**

## Discussion

ME/CFS is a potentially severe and disabling chronic illness affecting a large number of people in Ireland. Understanding its economic impact can help inform decisions relating to the provision of services and supports for those affected. A recent study for the European Network on ME/CFS (EUROMENE) concluded that the economic burden of ME/CFS in Europe is considerable, with scope for substantial savings through effective prevention and treatment ^10^. To date there has been no research on the economic impact of ME/CFS in Ireland. This paper represents the first step in addressing this gap. Using focus groups and thematic analysis, it identifies four inter-related themes, namely: (1) Healthcare barriers and costs; (2) Socioeconomic costs; (3) Costs of disability; and, (4) Carer-related costs.

Participants in our study identified a wide range of healthcare barriers and costs relating to ME/CFS. Getting a diagnosis was described as a particularly important and challenging issue, often stemming from a lack of understanding of, or disbelief about, the illness. For many of our study participants, this resulted in numerous consultations with a variety of healthcare professionals, often at considerable private expense. This experience is consistent with findings from one of the very few previous studies on ME/CFS in Ireland, which found a mean time to diagnosis of 3.7 years and a range of 0–34 years ^5^. It concluded that the priority for future service provision should be increased understanding and diagnosis of ME/CFS by the medical profession.

Delays in diagnosis is not an issue confined to Ireland. According to previous research from the UK, “a current problem regarding ME/CFS is the large proportion of doctors that are either not trained or refuse to recognise ME/CFS as a genuine clinical entity, and as a result do not diagnose it” ^34^. Another UK study, which included a survey of GPs’ attitudes and knowledge around ME/CFS, found that “despite the publication of guidance for GPs on CFS/ME, confidence with making a diagnosis and management was found to be low” ^35^. This is an important issue in the context of understanding the economic impact of ME/CFS, since it has been claimed that “improved understanding of the illness pathology, diagnosis, and management, may reduce costs, improve patient prognosis and decrease the burden of ME/CFS” ^28^. Furthermore, there is evidence that delayed diagnosis may be a risk factor for poor disease prognosis ^3^, while it could also have implications for qualifying for State supports. Without a diagnosis, many people will not be entitled to supports, increasing their out-of-pocket expenses.

Problems with healthcare professionals and services were not limited to diagnosis and again tended to result from scepticism about the condition or a lack of awareness and understanding. Often this led to a variety of healthcare costs for patients, such as paying extra for specialist consultant care instead of standard GP care. Previous research found that patient satisfaction with medical professionals was low in general and, as a result, patients opted for alternative or complementary forms of treatment, often driven by insufficient informational and emotional support from their doctors ^36^. Participants in our study also described turning to alternative therapies and treatments, which they described as very expensive, often following poor experiences with their doctors. A lack of effective traditional medical treatments for ME/CFS was also a reason. High levels of expenditures on alternative treatment have been found in other studies internationally ^7, 28^.

Disagreements over illness aetiology and treatment have previously been identified as problematic in the ME/CFS patient-doctor relationship ^36^. This was also a particularly important issue for some of the participants in our study who described being misdiagnosed with a psychological condition: “they told me it was in my head”. Participants also described experiences with what they believed to be inappropriate treatments, such as cognitive behavioural therapy (CBT) or graded exercise therapy (GET), which either did not work or exacerbated the illness. These experiences are consistent with international evidence. For example, a recent study from the US states that medical training is inadequate regarding the symptomatology, prognosis, and treatment for ME/CFS and that, as a result, “many physicians lack the appropriate level of knowledge about effective methods for ME and CFS symptom reduction and often suggest inappropriate treatments, such as increased exercise or psychiatric services” ^37^. In fact, it has been argued that the available evidence does not suggest that interventions such as GET and CBT are safe and risk-free for ME/CFS patients ^38^, and that CBT is not effective and should be downgraded to an adjunct support-level therapy, rather than a treatment ^39^. More generally, it has been suggested that “there is little scientific credibility in the claim that psycho-behavioural therapies are a primary treatment for this illness” ^40^.

There are a number of reasons why this is an important issue within the context of considering the economic burden of ME/CFS in Ireland, or indeed elsewhere. First, healthcare costs to both providers and patients often represent a substantial component of the total economic burden in COI studies. If patients are receiving appropriate clinically- and cost-effective care, then such costs should not necessarily be seen as problematic. But if patients are receiving inappropriate care or treatments, then this likely represents an inefficient allocation of scare resources. A second reason why this is relevant is that patient participants in our study described how they stopped seeking healthcare as a result of the problems they faced with healthcare professionals and services, including inappropriate diagnoses and treatments. As a result, estimates of direct healthcare costs may well be lower than what might be optimal i.e. if patients were receiving appropriate care. Third, some participants described how the care and treatments they received exacerbated their condition. This is particularly relevant in terms of considering economic costs in the context of disease prognosis and disease severity.

Overall, in terms of healthcare services, participants described a range of barriers they faced, which in many cases led to higher healthcare costs for them, as well as for the public healthcare system. They also described how the majority of tests and treatments they tried were of limited effectiveness, despite often being very expensive. As a result, participants in our study experienced and described relatively poor disease prognosis and this led to a range of other costs, including socioeconomic costs and costs of disability.

In terms of socioeconomic costs, participants described a range of adverse labour market outcomes, including a reduced ability or complete inability to work, as well as having to take early retirement. In estimating the economic burden of ME/CFS in a COI context, this is a particularly important consideration, since the most substantial component of the burden is likely to be the indirect costs that arise as a result of productivity losses ^10, 19^. For example, a recent Australian study found that of the estimated AUS$14.5 billion annual cost of ME/CFS, 70% was due to lost income ^28^. But while these productivity losses represent an important cost from a societal perspective, it is important to highlight there are also important personal implications from the inability to fully participate in the labour market. For example, participants described the negative impact this had on their income and on their general standard of living. Issues relating to a lack savings or a pension were also described, as were problems with debt and mortgage difficulties. Therefore, these are all important considerations in any future research examining the impact of ME/CFS on the economic well-being of those affected.

While participants described a range of direct healthcare costs, as well as indirect costs such as lost income, they also outlined a number of additional so-called costs of disability. These extra or hidden costs can arise as either one-off/irregular expenses or extra costs of day-to-day living and can lead to a substantial reduction in standard of living ^41, 42^. Respondents in our study described the extra expenses they faced in terms of home adaptations, aids, and appliances, as well as more regular costs, including extra spending on items such as heating, transport, cleaning, and childcare services. These are important considerations in any assessment of the economic impact of a condition such as ME/CFS at a patient level. This is because spending on such items means that disposable income that would otherwise be spent on everyday items associated with a higher standard of living is diverted to expenditures that would not ordinarily be faced. This leads to a greater likelihood that people with a disability, such as ME/CFS, live in poverty and/or deprivation ^43, 44^.

A related issue, and one described as particularly problematic by participants in terms of their economic well-being, was the lack of appropriate services and supports available to ME/CFS patients. Difficulties accessing these arose due to a lack of awareness of ME/CFS, as well as high levels of bureaucracy or so-called administrative burdens ^45^. This resulted in many participants going without necessary services and supports, with a substantial negative impact on their quality of life, or paying for them themselves, often at considerable cost. A systematic review of the expressed needs of people with ME/CFS found consistent evidence that substantial support is needed to rebuild lives ^46^. Our focus groups suggest that such support may be missing for many patients in the Irish setting.

In discussing the impact of ME/CFS in our focus groups, specific additional issues arose for two groups in particular, namely children and carers. Both groups are important to consider in analysing the economic burden of ME/CFS, for a number of reasons. For example, it has been claimed that the experiences of parents who care for sons or daughters with severe ME are rarely discussed within the literature ^47^. In our study, carer participants of children described how healthcare professionals were sometimes particularly reluctant to give an ME/CFS diagnosis, while it was also stated that children were particularly vulnerable to a psychological misdiagnosis and/or weren’t believed. These issues generally led to additional health-related costs. In terms of socioeconomic costs, an important issue raised by younger participants, or carers of children, was the impact of ME/CFS on educational outcomes. The illness had a particularly negative impact on the ability to participate in education, both at secondary and third-level, often with negative consequences for educational attainment. This is important within the context of considering the economic impact of a condition such as ME/CFS, since human capital theory in economics clearly shows a strong relationship between educational attainment and future labour market outcomes. In other words, the negative educational consequences of ME/CFS for children and younger adults likely reduces their future productivity and earnings, with consequences for the economic impact on both individuals and wider society.

ME/CFS was also found to have a range of direct and indirect impacts on informal carers in our study. For example, carer participants described how they often spent considerable time caring for family members with ME/CFS. Informal care time is an important constituent of COI studies that take a wider societal perspective, while it has been argued that emphasising the importance of paid work over unpaid work in traditional national accounting metrics can lead to inefficient policy decision making ^48^. Our focus groups suggest that informal carers of patients with ME/CFS make a valuable contribution in terms of the time they allocate to such activities and this should be included in any future COI study of ME/CFS in an Irish context.

In addition to time costs, there were also other costs or impacts on carers that should be considered. In our study, carer participants described the often considerable toll that caring took on their mental health, with some describing how they experienced, or came close to experiencing, burnout. The issue of so-called caring externalities has been identified in previous research ^49^, though it is important to acknowledge that health spillovers can arise for both caregiving and non-caregiving family members ^50^. Other negative impacts on carers described in our focus groups included reduced work and study possibilities arising from caring responsibilities.

### Limitations

This was a small-scale qualitative research study based on three focus groups of patients with ME/CFS and informal carers of patients with ME/CFS. Due to the nature and locations of our focus groups, they did not include patients who were, at the time, house-bound or bed-bound, e.g. patients with very severe ME/CFS. In addition, they did not include patients with very mild ME/CFS or patients who had fully recovered. Furthermore, despite attempting to have a diverse set of participants across a range of other dimensions, we did not have any male patient participants. However, four informal carer participants were carers of male patients with ME/CFS. In this context, it is important to note that as with all qualitative research, the aim of this study was to improve understanding, as opposed to generate fully generalisable results. Future quantitative research seeking to generate estimates of the economic impact of ME/CFS should be based on a representative sample of patients where possible.

## Conclusion

There is distinct lack of evidence on the economic impact of ME/CFS in Ireland, at both an individual and wider societal level, and this study represents a first step in addressing this research gap. Our focus group participants identified a wide range of costs relating to healthcare, socioeconomic outcomes, disability, and caring. These costs, and the reasons they exist, are consistent with international evidence and should be examined in more detail in future research. In particular, research examining the burden of ME/CFS on individuals and society could help improve understanding of the condition among healthcare professionals, policymakers, and the general public. It could also be used to better inform strategies to mitigate the burden and costs of ME/CFS ^10, 28^. However, in terms of undertaking such research, it is important to note that the relatively small amount of existing research on ME/CFS is in part a result of the lack of funding available in this field/area. For example, it has been shown that ME/CFS is more underfunded with respect to burden than any other disease in the US, with the illness receiving roughly 7% of that commensurate with disease burden ^51^. To date, there has been very limited research on ME/CFS in Ireland and this is something that should be urgently addressed. Dedicated funding for such research would therefore be very welcome by the ME/CFS patient community in Ireland.

## Data availability 

### Underlying data

There are no quantitative data associated with this article. The audio files and recording transcripts from our focus groups are private and confidential. In particular, as per the research ethics approval for this study, the following measures were undertaken in order to protect the confidentiality and anonymity of our participants:

- Only members of the research team will have access to data collected as part of this study.
- Each participant will be assigned a numeric identifier and/or pseudonym.
- The master file with participants’ names and pseudonyms will be stored in a secured locked cabinet in NUI Galway, separate from any other data collected.
- Audio recording will be stored on a password protected computer.

As a result, researchers seeking to access the underlying data (i.e. audio files and transcripts) will need to apply directly to the NUI Galway Research Ethics Committee for approval. The Committee can be contacted at ethics@nuigalway.ie. Should approval be granted, the authors are happy to facilitate access. Quotes reflecting the transcripts are available in the article itself.
