# Supplementary material for: Understanding the economic impact of myalgic encephalomyelitis/chronic fatigue syndrome in Ireland: a qualitative study
Source: HRB Open Res. 2020 Dec 4;3:88. [Version 1] doi: 10.12688/hrbopenres.13181.1 (PMC7898356; doi:10.12688/hrbopenres.13181.1)
Supplement: Peer review history

# Peer review: Reviewer response for version 1

**DOI:** 10.21956/hrbopenres.14309.r28827

**Published:** 2021-02-18

**Authors:** Suzanne Vernon

**Affiliations:** 1 The Bateman Horne Center of Excellence, Salt Lake City, UT, USA

This study used thematic analysis of focus group interviews to identify economic impact factors that effect ME/CFS patient partners and care providers living in Ireland. This study was implemented three ME/CFS patient partners and one academic. There were 3 focus groups in 3 separate regions of Ireland.

 The thematic analysis identified familiar themes that effect patients and their caregivers. Many of these themes are "hidden" for the reasons the authors so eloquently discuss in the paper. Perhaps the authors would consider renaming their paper to "identifying hidden factors of economic impact in ME/CFS". Then this approach could be used to quantify the "true" economic burden of ME/CFS. The authors are correct to point out that other economic impact studies (and corresponding papers) likely underestimate the true economic impact. The challenge will be translating some of these themes to a value. This will be a large, yet important undertaking. I think this aspect deserves a bit of discussion in the discussion.

 While determining economic impact is an important step in helping funding agencies and policy makers understand the disease burden and impact on the individuals and societies, these analyses don't necessarily translate into action. Notably, one of the first ME/CFS economic impact studies was done 15 years ago by a US federal agency. Yet it did not translate into increased funding or policy change. Sadly, as the authors point out, providers still don't seem to know what ME/CFS is or what to do with it despite the tremendous burden of disease and economic toll. The authors may want to consider a paragraph in the discussion describing how they want these results to be used to change the status quo.

Is the work clearly and accurately presented and does it cite the current literature?

Yes

If applicable, is the statistical analysis and its interpretation appropriate?

Not applicable

Are all the source data underlying the results available to ensure full reproducibility?

Partly

Is the study design appropriate and is the work technically sound?

Yes

Are the conclusions drawn adequately supported by the results?

Yes

Are sufficient details of methods and analysis provided to allow replication by others?

Yes

Reviewer Expertise:

Virology, Infectious Disease, Clinical Research, Public Health Epidemiology, Outcomes Research.

I confirm that I have read this submission and believe that I have an appropriate level of expertise to confirm that it is of an acceptable scientific standard.

# Peer review: Reviewer response for version 1

**DOI:** 10.21956/hrbopenres.14309.r28517

**Published:** 2020-12-09

**Authors:** Donald Staines

**Affiliations:** 1 National Centre for Neuroimmunology and Emerging Diseases (NCNED), Menzies Health Institute Queensland, Griffith University, Gold Coast, Australia

This small qualitative economics study on ME/CFS is an important contribution to understanding the impact of this debilitating illness in Ireland. The methodology is structured around interviews with 21 patients and carers (15 patients plus 6 carers) meaning that some subjective elements will be provided through this discourse approach. Further, the study acknowledges that housebound or severe patients were not included. Hence there are intrinsic shortcomings in the structure and conduct of the study. However these are acknowledged by the authors and these relative deficiencies are noted for more comprehensive methodological design in future studies. There was suitable geographic spread in the study for the three sites chosen.

 The findings are grouped into four broad categories: Healthcare barriers and costs, socioeconomic costs, costs of disability and carer-related costs. Delays in obtaining an appropriate diagnosis, sometimes a number of years, poor knowledge of the illness by professionals and lack of treatments were predominant themes which added to barriers in receiving proper care. Invariably these factors impact on economic well-being for employment opportunities for both patients and carers. Loss of social roles and impacts on relationships are often hidden but critical components of the overall burden of the illness. Problems such as disbelief of children with ME/CFS were identified and occur around the world. The impact on their education is often under estimated.

 Importantly the findings of this study echo other studies in terms of methodological approaches and key findings. Hence while of relatively small scale, its importance should not be underestimated. As the authors note, this is the first such study on health economics for ME/CFS in Ireland and is therefore to be welcomed to the literature. Economic costs to the health care system and the wider community are common findings in other larger studies. Quantification of all the findings in this study will likely be improved in subsequent studies and such studies should be encouraged. Certainly issues around diagnostic accuracy and prevalence have a significant bearing on economic impact studies and this is acknowledged by the authors. The article also notes that the study is an important starting point for further studies on socioeconomic impact investigations for ME/CFS in Ireland. The lack of attention to this illness in Ireland has translated to inferior funding for diagnosis, treatment and research.

 Overall the article is compelling reading for consumers and providers of health care as well as research funding agencies. The referencing of the article is comprehensive and appears appropriate for the nature of the study and its findings.

Is the work clearly and accurately presented and does it cite the current literature?

Yes

If applicable, is the statistical analysis and its interpretation appropriate?

Yes

Are all the source data underlying the results available to ensure full reproducibility?

Yes

Is the study design appropriate and is the work technically sound?

Yes

Are the conclusions drawn adequately supported by the results?

Yes

Are sufficient details of methods and analysis provided to allow replication by others?

Yes

Reviewer Expertise:

Public health medical and scientific research aspects of ME/CFS.

I confirm that I have read this submission and believe that I have an appropriate level of expertise to confirm that it is of an acceptable scientific standard.
